# Preserving Connection: The Vital Window of Speech Therapy Intervention in a Dementia Care Case Study

**DOI:** 10.1093/geroni/igaf122.176

**Published:** 2025-12-31

**Authors:** Collean Combs

**Affiliations:** University of New Hampshire, Durham, New Hampshire, United States

## Abstract

This case illustrates that delayed implementation of best practice for early and frequent opportunities to intervene in dementia care can significantly impact patients’ communication abilities as well as QoL for both patients and care partners. This situation underscores the need for systematic improvements in implementation science related to education and intervention in dementia care, emphasizing the importance of interdisciplinary management that includes SLPs. A 63-year-old man diagnosed with Alzheimer’s disease went 4.5 years without a speech-language consultation, despite declining cognitive linguistic abilities noted by his neurologist. Neither he nor his care partner received guidance on interventions, leading the care partner to independently seek services, self-referring to the clinic where a comprehensive cognitive-communication evaluation was completed. The evaluation revealed, despite severe cognitive impairment and notable word finding difficulties, the patient’s syntactic and grammatical structures were largely intact. Although the foundation of language was preserved, the patient lacked compensatory communication strategies during these challenges, which hindered his ability to convey his overall message effectively, resulting in frequent communication breakdowns. These findings led to a patient-centered intervention designed to equip both the patient and their care partner with tools to address communication breakdowns. This included the implementation of cognitive-communication strategies and care partner training, providing specific methods to enhance communication and reduce frustration. This comprehensive approach improved the patient’s overall functional communication, resulting in a reported increase in QoL for both the patient and care partner, highlighting the value of early referrals to speech-language services in dementia care.

